# Feasibility and Engagement of a Mobile App Preparation Program (Kwit) for Smoking Cessation in an Ecological Context: Quantitative Study

**DOI:** 10.2196/51025

**Published:** 2024-10-02

**Authors:** Luz Adriana Bustamante Perez, Lucia Romo, Oulmann Zerhouni

**Affiliations:** 1 Laboratoire EA 4430-Clinique Psychanalyse Developpement, Department of Psychology, University of Paris Nanterre Nanterre France; 2 Inserm-Le Centre de Recherche en Epidémiologie et Santé des Populations 1018 UPS Paris France; 3 Université de Rouen Rouen France

**Keywords:** smoking cessation, digital intervention, behavior change techniques, attrition rate, mobile app, preparation program, motivation, quit smoking, ecological settings, mobile phone

## Abstract

**Background:**

Mobile health apps can facilitate access to effective treatment and therapeutic information services. However, the real-world effectiveness of mobile apps for smoking cessation and their potential impact in everyday settings remain unclear.

**Objective:**

In an ecological context, this study aimed to estimate the engagement rate of a mobile app–based smoking cessation preparation program and its potential impact on users’ willingness, ability, and readiness to quit smoking.

**Methods:**

A total of 2331 “organic users” (ie, users who discover and install a mobile app on their own, without any prompts) chose 1 of 2 program versions of the mobile app (Kwit): the basic version or the premium version. Both versions were identical in design, with 4 more evidence-based content items and strategies in the premium version. Outcomes were analyzed based on automated data registered in the app (engagement rate, motivation to quit, motivation type, motivation levels, and satisfaction level). Mann-Whitney and *χ*^2^ tests were used to compare the results of both groups.

**Results:**

As expected, in the ecological context, a high dropout rate was observed at different moments. A significant difference was observed between the 2 versions (n=2331; *χ*^2^_1_=5.4; *P*=.02), with a proportionally higher engagement rate in the premium version (premium=4.7% vs basic=2%). Likewise, differences were also observed between the 2 groups in terms of reasons to quit (n=2331; *χ*^2^_4_=19; *P*≤.001; V=0.08), motivation type (n=2331; *χ*^2^_7_=14.7; *P*=.04), and motivation level. Users of the app’s premium version more frequently reported “well-being” (23.3% vs 17.9%) and “planning a pregnancy” (7.4% vs 4.4%) as their primary reasons for quitting smoking compared to those with the basic version. Moreover, they reported being more likely to be driven in the smoking cessation process by intrinsic motivation (premium=28% vs basic=20.4%), as well as feeling significantly more willing (*z* score=156,055; *P*≤.001; Cohen *d*=0.15), able (*z* score=172,905; *P*=.04; Cohen *d*=0.09), and ready (*z* score=166,390; *P*=.005; Cohen *d*=0.12) to stop smoking than users who had the basic version before completion of the preparation program. Among participants who finished each version of the program (premium: 9/189, 4.8%; basic: 47/2142, 2.19%), significant improvements in motivation levels were observed in both groups, although in different areas for each group (willingness levels for the premium group and ability for the basic group).

**Conclusions:**

These results suggest that even in ecological contexts where engagement rates are meager, the Kwit preparation program can address ambivalence by increasing willingness to change, self-confidence, and readiness to quit among its users, especially those who feel less able to do so. Further development and evaluations are needed to better understand determinants for regular mobile health apps.

## Introduction

### Tobacco Consumption

The World Health Organization (WHO) considers smoking an epidemic that affects >1 billion people worldwide. It is a public health issue for many countries because tobacco use is one of the leading risk factors for a significant number of deaths and disabilities worldwide, and although preventable, the economic and social costs of disease burden remain very high [1]. On the basis of WHO recommendations, France’s national tobacco reduction program successfully addressed tobacco consumption, as the quit rate has been decreasing significantly since implementing several tobacco control policies in 2014 (ie, the reimbursement of approximately EUR €150 [US $166] per year for nicotine replacement products, monitoring of health warnings, media restrictions, and taxation of cigarette packs) [2].

Although all these policies have had some success in increasing smoking cessation, the long-term abstinence rate is still extremely low despite 60% of worldwide smokers expressing their willingness to quit. In France, at least 29.9% of daily smokers tried to stop for at least a week the previous year without long-term success [2]. In addition, results from double-masked clinical trials show that the willingness to quit and actual reductions in smoking behavior are limited by the content of nicotine present in cigarettes, even in participants who were not initially interested in quitting [3,4]. Complex factors must be considered for long-term abstinence, such as the social influence of marketing misinformation about tobacco consumption, nicotine pharmacokinetics, everyday cue conditioning, withdrawal symptoms, and a comprehensive discussion of treatment options and goals [5].

### Treatment for Smoking Cessation

Multiple treatment approaches have been explored because of the complexity of tobacco dependence. To date, 2 major treatment categories have been used to treat tobacco use disorder: pharmacological treatments (eg, nicotine replacement therapy, bupropion, and varenicline) and nonpharmacological approaches (eg, motivational interviewing, cognitive and behavioral therapies, and acceptance and commitment therapy) [6]. In addition, new treatment goals have been proposed as alternatives to total and abrupt abstinence. These include gradually reducing cigarette consumption or using methods like snus and vaping to lower the risks associated with smoking, particularly for individuals with high nicotine dependence [4,5].

Both WHO and the French Ministry of Health recognize the impact of mobile health (mHealth) by promoting communication on social networks and using new technologies (eg, helplines, government mobile apps, and specific websites) to facilitate access to information and, thus, health care for smoking cessation [7,8].

### mHealth Apps for Smoking Cessation

#### Overview

mHealth refers to emerging technologies for accessing health and medical services through mobile devices [9]. This includes mobile eHealth apps (known as mHealth apps), which are software that provide health and wellness services, designed exclusively for mobile devices (smartphones and tablets) [10]. With nearly 70% of the world’s population having a smartphone and access to the internet, mHealth apps can promote access to smoking cessation treatments [11]. mHealth apps can facilitate access to effective treatments and therapeutic information services by (1) adapting and translating “active principles” (the term used to refer to the various strategies and practices of evidence-based behavioral and cognitive therapies) into a digital format; (2) facilitating communication with health professionals, allowing a more flexible and personalized relationship (chat and social groups); and (3) encouraging a sense of responsibility and commitment to one’s health through “nudges,” such as positive reinforcement through messages or notifications, habit tracking, regular feedback, and audiovisual support [12].

Despite the many benefits of using mobile apps for smoking cessation, research is still in its infancy. It faces specific challenges: the quality of content, the range of potential uses, and engagement with mHealth apps for smoking cessation.

#### Content Quality of Mobile Apps for Smoking Cessation

According to a systematic review and meta-analysis, there is a relationship between the number of cognitive and behavioral techniques used in smoking cessation programs and short- and long-term effectiveness [9]. However, according to a recent review of the content analysis of mobile apps for smoking cessation in France, in 2020, (1) the average quality of mobile apps was 3.5 out of 5 (median 3.1; range 1-3) based on the Mobile App Rating Scale, (2) most of the apps made little use of the “active ingredients” of evidence-based therapies (between 4 and 38 out of 93 indexed cognitive behavioral therapy [CBT] techniques), and (3) there was a lack of information that delivers proper advice regarding the use of approved pharmacotherapy or the implementation of behavioral techniques specific to helping people prepare for smoking cessation[10]. In contrast, only some apps have been extensively studied and made available in France [11]; however, they still need to be presented using a proper taxonomy of behavior change techniques to facilitate further research on their mobile app content quality.

#### The Range of Potential Uses of an mHealth App

Current studies primarily address smoking cessation mobile apps as self-help material used as a stand-alone treatment by their users [12-14], not focusing on other treatment goals of smoking cessation interventions such as facilitating risk reduction and relapse prevention [5,15,16]. Moreover, mobile apps can also be used as a complementary tool that enables continual accompaniment of the patient throughout their treatment by health professionals. The transtheoretical change model framework developed by framework of Prochaska and Prochaska [17] will be used to illustrate this purpose.

This model states that a long-term change (ie, smoking cessation) results from different stages. Each stage has specific challenges; once addressed successfully, the person can continue their change journey by following the next stage. The first stages of change (precontemplation, contemplation, and preparation) are about raising awareness of the target behavior and creating an action plan by identifying the benefits of quitting, decreasing the perceived difficulties of change, and acknowledging the pharmacological and psychological therapy. Hence, mobile apps can highlight therapeutic information on dependency and how pharmaceutical aids work. Mobile apps can also be used as an ecological momentary assessment tool through which it is possible to monitor cravings in real time to collect information that helps to formulate hypotheses on the precipitating and maintaining factors that lead to tobacco consumption [18]. On the basis of the previous assessment, it is possible to individualize therapeutic interventions by understanding the individual experience of craving through repeated and ecological measures [15].

In the action phase, the person can change the target behavior [17]. Therefore, to manage cravings differently instead of smoking, a person can experiment with new methods that can be transitional and complementary, such as the use of pharmacological (ie, nicotine replacement therapy or medications) and psychological substitute strategies (ie, avoiding a specific place or person). In this phase, mobile apps can be a tool that prompts experimentation of new strategies by giving different types of feedback on behavior (ie, monitoring nicotine consumption, meditation exercises, distraction activities, etc) as well as being a motivational levier that highlights the benefits of smoking cessation or its reduction in different aspects of the user’s life (health benefits and savings).

In the maintenance phase, “relapse” is perceived as the norm and not the exception, so it can be a learning opportunity in which the goal is to anticipate and prevent high-risk situations as well as navigate through the moment of the onset of craving by creating a relapse prevention plan [17]. Mobile apps can be a tool that helps monitor emotional changes and track withdrawal symptoms, both very common to relapse. On the basis of previous use, the mobile app can also provide feedback indicating treatment effectiveness and the risk of relapses.

As a result of these insights, Kwit app—a French mobile app initially conceived for maintaining smoking cessation—released the Kwit’s 9-step preparation program for smoking cessation (9s-Kwit’s program) in July 2021*.* A new feature specifically designed for users self-identifying as being in the preparation phase of smoking cessation according to the transtheoretical model of change developed by Prochaska and Prochaska [17], this program is based on cognitive and behavioral therapy for smoking cessation as well as dialectical and behavioral therapy, motivational interviewing, and self-determination theory.

#### Engagement With Digital Interventions Such as mHealth Apps for Smoking Cessation in Real Settings

As in any treatment protocol, “adherence to therapies is an important determinant of treatment success” [19]. The smoking cessation intervention attrition rate could be as high as 77% [20]. Conversely, mobile apps for chronic diseases face similar challenges as other mHealth apps, with attrition rates ranging from 43% (95% CI 16-63) in randomized controlled trials to 49% (95% CI 27-70) in observational studies [21]. To date, the dropout rate of some smoking cessation apps tested in a controlled scenario could vary from 19.3% after 28 days of use [22] to 39.5% after 87.3 days [23]. However, it remains to be seen what the attrition rate of smoking cessation mobile apps in ecological contexts would be [24-27], particularly with organic users. In this study, “organic users” or “organic installs”—terms primarily used in mobile app marketing—refers to individuals who discover and install a mobile app on their own, without any influence from paid marketing. Crucially, for the purposes of our research, these organic users are characterized by their autonomy to download the app and pursue smoking cessation. This autonomy distinguishes them significantly from users who participate in studies for compensation or who are mandated to use the app by health care institutions. Therefore, it is hypothesized that organic users will exhibit different behaviors and levels of engagement in smoking cessation compared to their nonorganic counterparts.

In conclusion, even if an app includes all the therapeutic guidelines, it can only be effective if used regularly [10,24,27]. Understanding how users engage periodically with their health treatment is challenging for mHealth apps [24,27,28]. Therefore, it is essential to identify and assess the main factors that favor using mobile apps for smoking cessation. To this end, the various research methods should ensure ecological validity in a design of evaluation setup that matches the user’s real work context [29] and ensures that the behavioral responses obtained represent the natural behaviors of people who want to quit smoking with the help of a mobile app [30].

The aims of this study were, therefore, to (1) explore, in an ecological context, the engagement rate of a 9s-Kwit’s program on a mobile app and (2) estimate the impact of the program on the motivation level among users who finished the program.

We hypothesized that (1) given the ecological context of this study, we will observe a remarkably high dropout rate between 80% and 95% of the users participating in the study. In addition, we sought to examine some key factors’ role in the Kwit program’s engagement rate. Specifically, (2) we hypothesized that the attrition rate within the intervention could be explained by the version of the program (basic and premium), the lack of relevance and understandability of the program content, the primary motivation to quitting smoking (ie, “money” or “family”), the lower levels of internalization to stop smoking (external motivation), and the lower level of motivation to quit before starting the program. Finally, we hypothesized that (3) users’ perception of their willingness, ability, and readiness to stop smoking will significantly improve after finishing the 9s-Kwit’s program*.*

## Methods

### Overview

This observational study was conducted from July 4, 2021, to July 28, 2021, in an ecological context. It represents the natural behaviors of people who downloaded and used the app to prepare for smoking cessation. In detail, this ecological context includes the following (1) the participants were organic users (users that installed the app because of their search results and without having encountered paid advertisements before onboarding); (2) only information essential for the everyday use of the mobile app was collected, and only data required for the study were exported to be analyzed (ie, no demographic data were collected before onboarding); (3) no compensation was offered to the users for their participation in the study; and (4) they checked a nonpreemptive box to give their consent to the collection and analysis of data related to their use of the app for research and improvement purposes. If an organic user chooses not to allow Kwit app to use their data for research, this preference is recorded in the server. This choice does not affect the quality of the services offered by the Kwit app. All users, regardless of their data use choice, have the option to access the premium version of the app. This ensures that there is no ethical compromise in the provision of services.

### Ethical Considerations

The local French Ethical Committee (South-East) validated the protocol study on March 25, 2021. The research identification number is 2020-A02733-36, and the committee’s reference is CPP 20.10.02.44945 [31]. This study adheres to the ethical principles outlined in the Declaration of Helsinki. This feasibility study is part of a prospective study registered on April 8, 2022 (ClinicalTrials.gov NCT05318651). It aims to identify critical determinants of smoking cessation mobile app use among smokers seeking to quit.

### Participants

Participants were aged at least 18 years and had a compatible smartphone (iOS 13.3 and above) with regular internet service. In total, 2 groups of participants were followed according to the type of program version they had access to, basic or premium.

Before participating in the program, participants were required to create an account that pseudonymized their personal information, including billing information where applicable. The Kwit app offered 2 versions: premium and basic. The choice between them was influenced by whether the participant opted for a 7-day free trial.

The premium version was available to users who opted for the free trial and provided full access to all features of the program. In contrast, the basic version limited the user’s access to only 4 levels of the program (steps 0, 1, 2, and 8). It is important to note that both versions visually presented the same program dashboard screen to ensure a consistent user experience. The user journey leading to the 9s-Kwit’s program dashboard is detailed in Multimedia Appendix 1.

In terms of pricing structure, the premium version of the Kwit app was offered through a 7-day free trial during the study period from July 4, 2021, to July 28, 2021. This trial period gave users the flexibility to discontinue the service if they wished. After the trial period, if the subscription was continued, it was considered an agreement to a monthly membership fee of EUR €9 (US $9.96). This pricing was strategically set to approximate the cost of a pack of cigarettes in France at the time, aligning the cost of the app with a tangible smoking expense and making the value proposition relatable and practical for users.

### Intervention

The 9-step Kwit’s program was developed after 2 years of research and development, as part of a PhD thesis, in collaboration with the University of Paris Nanterre. The content of this program was defined and coordinated by the cognitive behavioral psychologist and PhD student to be then executed by a team composed of a user experience designer, 3 software developers with at least 10 years of experience, a researcher in public health and clinical research, and 4 interns with a psychology master’s degree. The 9s-Kwit’s program has been developed for commercialization and designed for direct delivery as a mobile app intervention on Apple and Google Stores. This program is based primarily on cognitive and behavioral therapy for smoking cessation but is also inspired by dialectical and behavioral therapy, motivational interviewing, and the self-determination theory.

The 9s-Kwit’s program comprises 9 steps from step 0 (s0) to step 8 (s8). Through different activities, each step aims to explore a specific aspect of tobacco use that has already been identified as essential to consider in treating smoking cessation [9,32]. A presentation of the 9s-Kwit’s program’s home screen, a summary of its contents, and an example of the activity screen per step are presented in Multimedia Appendix 2.

The presentation of the activity starts as follows: each activity starts with an informative screen that introduces the activity’s main goals, then the activity itself, and ends with a feedback screen (Multimedia Appendix 3). When the user finishes an activity, the next one is unblocked. Different types of feedback coexist to inform the user that an activity is completed: (1) the color of the activity title changes (ie, in s0 from gray to blue), (2) information about the date and hour is presented instead of the average duration of the activity, (3) the icon next to the title activity changes from a lock to an arrow to show that the next activity is then accessible, and (4) the activities that are already done have a summary card if you click on it. Once all activities of the same step are completed, the next step is unlocked.

The initial step (s0) consists of becoming aware of one’s motivation to engage in a change process, but to change the target behavior, motivation alone is not enough; it is crucial to get out of “self-pilot” mode and adopt an “observer attitude” to understand the association between the target behavior, its antecedents, and its consequences. Therefore, in the first step (s1), users are encouraged to monitor their behavior and create a baseline of the contexts and intensity of cravings and the inner strategies that have helped them cope. The behavioral baseline provides users with an objective measure that assists them in recognizing patterns of cravings and helpful strategies. The second step (s2) focuses on the different components of dependency, and by using the Horn scale, it is possible to explain how opposing stimuli can induce people to smoke (ie, relaxation vs stimulation). On the basis of the results, users receive advice on new behavioral strategies. The third step (s3) proposes that users categorize cravings according to their short-term benefits (pleasure or relief) and then some mindfulness exercises. The fourth step (s4) defines a goal and a road map adapted to the user’s resources. Steps 5, 6, and 7 teach new coping strategies for the 3 dependence types (s5: environmental, s7: psychical, and s7: psychological). The final step (s8) invites users to define a quit date and acknowledge the cognitive barriers to quitting. The program encourages being kind to oneself when slips and relapses occur and celebrating each small step toward the desired change. None of the program versions used in this study have the gamification layer where users earn points at the end of each activity, have their avatar, or any educational reading content or community layout that now exists in the platform. Table 1 details all activities of each of the 9s-Kwit’s program according to the taxonomy on behavior change techniques developed by Michie et al [33].

**Table 1:** Overview of the behavioral and cognitive techniques (BCTs) and goals presented by step (s) and activity (a) of the 9s-Kwit’s program based on the taxonomy developed by Michie et al [33].

ABS^a,b^	Type of activity	BCT number and label^a^
s0a1	Identify main reason to quit: health, well-being, economy, family, and planning a pregnancy	1.9 Commitment
s0a2	Identify the level of willingness, ability, and readiness to quit smoking using a visual analog scale	1.9 Commitment
s0a3	Define a landmark that recalls reason to quit when craving arises	1.9 Commitment
s0a4	Identify the degree of internalization of abstinence motivation using the French Smoking Cessation Motivation Scale	1.9 Commitment
s1a1	Introduction of the “plus bottom”: craving arousal monitoring for 24 hours: context, intensity, and action (let it go or smoke)	2.1 Self-monitoring of the behavior
s1a2	Mindfulness exercises (3 minutes) focus on observing automatic responses when cravings arise, encouraging an observer’s attitude	8.2 Behavior substitution
s2a1	Horn scale: physical, psychological, and behavioral dependency assessment	2.7 Feedback on outcome of behavior
s2a2	Identify the relationship with smoking through an open self-questionary about when the behavior installs and the reason it maintains in the time	4.2 Information about antecedents
s3a1	Identification of the most difficult craving to overcome in the journey and it impacts on the body and cognition	5.3 Monitoring of emotional consequences
s3a2	Classify cravings into anchored (routine) and reflex (contextual) types	4.1 Instruction on how to perform a behavior
s3a3	Mindfulness exercises (3 minutes)	8.2 Behavior substitution
s4a1	Assessment of current resources available to execute an action plan	1.4 Action planning
s4a2	Three types of experiences were proposed to be completed within 24 hours based on S4a1 for craving management: (1) “Act consciously” mindfulness exercises: for those not willing to stop smoking; (2) “Choosing your cravings”: based on S3a1, users anticipated strategies to reduce “anchored” cravings; and (3) “Overcoming all cravings:” users anticipated craving management strategies for all cravings	1.1 Goal setting behavior and outcome
s4a2	Three types of experiences were proposed to be completed within 24 hours based on S4a1 for craving management: (1) “Act consciously” mindfulness exercises: for those not willing to stop smoking; (2) “Choosing your cravings”: based on S3a1, users anticipated strategies to reduce “anchored” cravings; and (3) “Overcoming all cravings:” users anticipated craving management strategies for all cravings	11.1 Regulation by pharmacological support
s5a1	Assess nicotine dependency with the Fagerström Test for Nicotine and give proper feedback about dependency level and offer recommendations for pharmacological therapy options that could complement the app, if necessary	8.2 Behavioral substitution
s6a1	Introduction of new features of the “plus bottom”: tracking nicotine substitutes or the vape consumption, mindfulness exercises, drinking water, and breathing exercises. Advise on how to avoid exposure to specific social and contextual cues	12.1 Restructuring the physical and social environment
s7a1	Identify barriers to quitting, such as learned helplessness, fear of withdrawal symptoms, and automatic behaviors related to craving management	13.3 Incompatible beliefs
s7a2	Recognize a behavior as it is and not as part of user’s identity	13.4 Valued self-identity
s8a1	Identifying barriers to quit and propose some solutions to overcome it: the fear to stop smoking (eg, weight, stress, and concentration)	8. Behavior rehearsal
s8a2	Identify the level of willingness, ability, and readiness to quit smoking using a visual analog scale of s0a1 and give proper feedback	13.3 Incompatible beliefs
s8a3	Recognize old behavior goals and consolidate experience through feedback. Define a future quit date	1.11 Review behavior goals

^a^This program content is created based primarily on cognitive and behavioral therapy for smoking cessation as well as dialectical and behavioral therapy, motivational interviewing, and self-determination theories.

^b^Activity by step=first activity of the step 0 (s0a1).

### Outcome Variables and Measurements

In total, 4 different types of measurements were used: ecological momentary assessment to measure engagement rate and version of the program (“a method of data collection whereby a record is made each time a predefined event occurs” [18]); a visual analog scale (VAS) to measure user’s perception of their willingness, ability, and readiness to quit smoking (the VAS slider ranged from 0 to 10); a Likert-type scale to measure the internalization degree to stop smoking, which can range from none (amotivation) to completely internalized (intrinsic motivation); and a multiple-choice question to measure user’s relevance and understandability of the program content and the users’ main reason to quit. Examples can be found in Multimedia Appendix 3.

#### Engagement Rate Toward the 9s-Kwit’s program

The engagement rate is the ratio of users who completed the program from the first use (step 0) to the last activity proposed in 9s-Kwit’s program (step 8).

#### Motives for Quitting Smoking

On the basis of previous studies on reasons for quitting smoking [34-36], users were asked to choose 1 of 5 reasons: health, wellness, money, family, and planning a pregnancy, using the following statement: *“*My main reason for quitting smoking is...” (Multimedia Appendix 3).

#### Motivation Level: The Willingness, Ability, and Readiness to Quit Scale

Using a VAS, users rated their perception of their willingness, ability, and readiness to quit smoking at the beginning (S0) and the end of the program (S0). For this purpose, each question was presented on a single screen and in the following order: (1) “To what extent this change is a priority for you right now?” (*willingness*), (2) “To what extent are you confident in your ability to change right now?” (*ability*), and (3) “To what extent do you feel ready to take action?” (*readiness*). This way, users could move a slider from 0 (lowest) to 10 (highest) on each screen (Multimedia Appendix 3). For statistical analysis purposes, scores <3 are defined as “low,” scores between 4 and 7 are defined as “moderate,” and scores >7 are defined as “high.”

#### Nature of Motivation to Quit Smoking

The French Smoking Cessation Motivation Scale (F-SCMS) is a self-reported measure based on the self-determination theory, demonstrating good internal consistency (α=.86; ωh=0.7; ωt=0.89) and content validity (common-fit index=0.905, standardized root mean square residual=0.045, and root mean square error of approximation=0.087). The 18 items are divided into 6 subscales (each composed of 3 items) corresponding to the degree of internalization that participants have toward quitting smoking. The subscales are presented from no internalization to complete internalization of a behavioral change process: (1) amotivation, (2) external, (3) introjected, (4) mixed, (5) identified, (6) integrated, and (7) intrinsic motivation. We have previously validated the scale (F-SCMS) used in this study and explained the internalization process in a separate publication [37].

The scale was presented as follows within the app. Each screen was composed from top to bottom in the following order: (1) the statement “Right now,” (2) the item appearing as a card, and (3) the list with 5 Likert-type response options ranging from 1 (does not match at all) to 5 (matches exactly). Once the user answered all the items, they could receive predefined feedback corresponding to their degree of internalization [37].

#### Perceived Content Relevance of s0

The last activity of s0 of the program was the evaluation by the users of the comprehensibility and relevance of the contents presented during this step. The question was presented as “You have completed Step X. How would you define it?*”* with the following 3 options: (a) *understandable and relevant*, (b) *understandable but irrelevant*, and (c) *not understandable and irrelevant.*

### Statistical Analysis

We performed descriptive analyses to characterize the 2 groups’ baseline samples and outcomes of interest. We conducted the Mann-Whitney test and a *χ*^2^ test of association to compare differences between both groups at the beginning of the program in terms of the main reason to quit, the motivation level to quit smoking, the nature of the motivation, and the perceived content relevance at the beginning of the program. We conducted a Student *t* test (2-tailed) to estimate the program’s impact on the motivation levels of the program completion for each group. For each test, a *P* value <.05 will indicate statistical significance. Analyses were conducted using Jamovi V2.3.8.

## Results

### Overview

A total of 2331 users started the preparation program. Overall, 91.89% (2142/2331) of the initial sample used the basic version of the program, and 8.1% (189/2331) used the premium version. As a reminder, the app’s premium version allowed access to each program step’s activities (from s0 to s8), whereas participants with the basic version had access only to s0, s1, s2, and s8 activities.

Table 2 presents the distribution of participants of both versions of the program in terms of (1) motives for quitting smoking, (2) motivation level to quit smoking, (3) motivation nature, and (4) perceived content relevance of step 0. In general, participants’ main reason for quitting smoking was health (1173/2331, 50.32%), followed by savings (450/2331, 19.31%), well-being (428/2331, 18.36%), family (172/2331, 7.38%), and planning a pregnancy (108/2331, 4.63%). At baseline, 80.65% (1880/2331) of the total sample was moderately motivated to quit smoking according to the Willingness, Ability, and Readiness to Quit (WAR) scale mean score, and 50.45% (1176/2331) fell into the 2 highest categories of internalization level for smoking cessation—integrated motivation (687/2331, 29.47%) and intrinsic motivation (489/2331, 20.98%)—according to the F-SCMS scale. Upon completion of step 0, the content of this step was considered by 71.55% (1668/2331) of the users as understandable and relevant (option A), by 10.98% (256/2331) as understandable but irrelevant (option B), by 0.82% (19/2331) as incomprehensible and irrelevant (option C), and 16.65% (388/2331) of the initial sample did not answer the question.

**Table 2:** The quantity and proportion of participants engaged in each activity of step 0^a^ (motives for quitting smoking, level of motivation to quit smoking, nature of motivation, and perceived content relevance).

		Total (N=2331), n (%)	Basic step 0 (n=2142), n (%)	Premium step 0 (n=189), n (%)	Chi square (*df*)		*P* value	
**s0a1^b^: Motives for quitting smoking**						19 (4)		.001
	Health	1173 (50.3)	1073 (50)	100 (52.9)				
	Well-being	428 (18.4)	384 (17.9)	41 (23.3)				
	Savings	450 (19.3)	434 (20.3)	14 (8.5)				
	Family	172 (7.4)	157 (7.3)	14 (7.9)				
	Planning a pregnancy	108 (4.6)	94 (4.4)	14 (7.4)				
	—^c^	0	0	0				
**s0a2^d^: Level of motivation to quit smoking**						6.48 (3)		.09
	Low	144 (6.2)	140 (6.5)	4 (2.1)				
	Moderate	1880 (80.7)	1724 (80.5)	156 (82.5)				
	High	235 (10)	212 (9.9)	23 (12.2)				
	—	72 (3.1)	66 (3.1)	6 (3.1)				
**s0a4^e^: Nature of motivation to quit smoking**						14.7 (7)		.04
	Amotivation	81 (3.5)	78 (3.6)	3 (1.6)				
	External	224 (9.6)	205 (9.6)	19 (10.1)				
	Introjected	88 (3.8)	82 (3.8)	6 (3.2)				
	Mixed	250 (10.7)	228 (10.6)	22 (11.6)				
	Identified	155 (6.6)	138 (6.4)	17 (9)				
	Integrated	687 (29.5)	635 (29.6)	52 (27.5)				
	Intrinsic	489 (21)	436 (20.4)	53 (28)				
	—	357 (15.3)	340 (15.9)	17 (9)				
**s0: perceived content relevance**						9.70 (3)		.02
	Option A^f^	1668 (71.6)	1516 (70.8)	152 (80.4)				
	Option B^g^	256 (11)	238 (11.1)	18 (9.5)				
	Option C^h^	19 (0.8)	19 (0.9)	0				
	—	388 (16.6)	369 (17.2)	19 (10)				

^a^s0: first step of 9s-Kwit’s program.

^b^s0a1: first activity of step 0.

^c^Not applicable.

^d^s0a2: second activity of step 0.

^e^s0a4: fourth activity of step 0.

^f^Indicates understandable and relevant.

^g^Indicates understandable but irrelevant.

^h^Indicates not understandable and irrelevant.

### Motives for Quitting Smoking

According to the *χ*^2^ test of association and Cramér value (n=2331; *χ*^2^_4_=19; *P*≤.001; V=0.08), users of each version of the program have reported proportionally different reasons for quitting smoking. Those who choose to get the premium version of the program were less likely to choose savings as their main reason to quit smoking (premium=8.5% vs basic=20.3%) but more likely to choose the well-being (premium=23.3% vs basic=17.9%) and planning a pregnancy (premium=7.4% vs basic=4.4%) options.

### Level of Motivation to Quit Smoking

Concerning the total score of the WAR scale, 3 levels of motivation were calculated based on the average score of 3 subscales: low (0-3), medium (4-7), and high (8-10). Most users (1880/2331, 80.7%) reported a moderate motivation to quit smoking at the beginning of the preparation program (s0; 1880/2331, 80.65%; mean 5.85 out of 10, SD 1.68). There was no significant difference between basic (n=2076) and premium (n=183) users when comparing these 3 categories (n=2331; *χ*^2^_3_=6.48; *P*=.09) at s0a2. Nevertheless, when the ordinal data were compared using the Mann-Whitney test, the 2 groups differed significantly but with a very slight effect within the total score and every subscale. Users who had the premium version of the app reported feeling significantly more willing (*z* score=56,055; *P*≤.001; Cohen *d*=0.15), capable (*z* score=172,905; *P*=.04; Cohen *d*=0.09), and ready (*z* score=166,390; *P*=.005; Cohen *d*=0.12) to stop smoking than users who had the basic version before completion of the preparation program.

### Nature of Motivation to Engage With a Smoking Cessation Process

The internalization degree of motivation to quit smoking differs significantly between basic and premium users according to the *χ*^2^ test of association (n=2331; *χ*^2^_7_=14.7; *P*=.04). Premium users are less likely to score in the “amotivated” profile (premium=1.6% vs basic=3.6%) and more likely to be driven by intrinsic motivation (premium=28% vs basic=20.4%) to engage in a smoking cessation process. In addition, the proportion of participants for whom no answer was received is lower in the premium group than in the basic group (premium=17/183, 9% vs basic=340/2076, 15.9%).

### Perceived Content Relevance of s0

The content assessment of s0 differs between premium and basic users according to the *χ*^2^ test of association (n=2331; *χ*^2^_3_=9.7; *P*≤.02), and by contrast, there are fewer premium users for whom a response is missing (–7.2%).

### Engagement Rate Toward the 9s-Kwit’s program for Smoking Cessation in an Ecological Context

#### Overview

**Figure 1:**
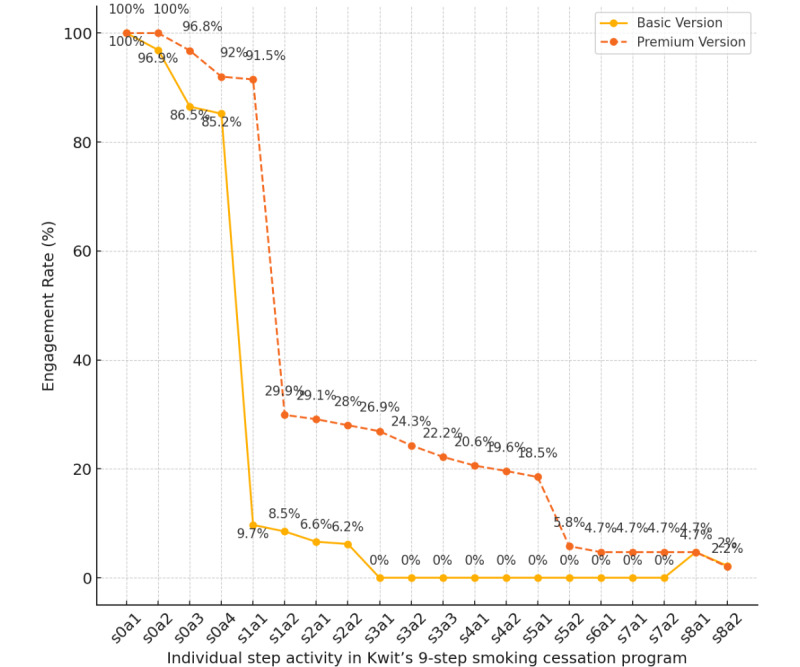
Engagement rate of the basic and the premium sample by activity for each step of the 9s-Kwit’s program. a: activity; s: step.

As shown in Figure 1, the engagement rate with the program drops significantly at 3 different moments. The biggest dropout concerns both samples (basic and premium) in between activities s0a4 and s1a1 (basic=–90.3% and premium=–70.1%). The second dropout was observed in the basic user sample between s2a2 and S8a1 (–4.2%), and the third dropout was within the premium sample from S4a2 to S5a1 (–12.5%). The proportion of participants who started each program activity is detailed in Table 3.

**Table 3:** Proportion of participants who completed each activity of the 9s-Kwit’s program.

Activities by steps^a^	Total sample started, n (%)	Basic sample started, n (%)	Premium sample started, n (%)	Chi-square test of association and Cramér value (*df*)	*P* value
s0a1	2331 (100)	2142 (100)	189 (100)	—^b^	—
s0a2	2259 (96.9)	2076 (96.9)	183 (96.8)	—	—
s0a3	2238 (96)	1853 (86.5)	174 (92)	—	—
s0a4	1998 (85.7)	1825 (85.2)	173 (91.5)	7.8 (1)	.005
s1a1	265 (11.4)	209 (9.7)	56 (29.9)	68.1 (1)	≤.001
s1a2	236 (10.1)	181 (8.5)	55 (29.1)	—	—
s2a1	195 (8.4)	142 (6.6)	53 (28)	—	—
s2a2	184 (7.9)	133 (6.2)	51 (26.9)	—	—
s3a1	46 (1.9)	0	46 (24.3)	—	—
s3a2	42 (1.8)	0	42 (22.2)	—	—
s3a3	39 (1.6)	0	39 (20.6)	—	—
s4a1	37 (1.6)	0	37 (19.6)	—	—
s4a2	35 (1.5)	0	35 (18.5)	—	—
s5a1	11 (0.5)	0	11 (5.8)	—	—
s6a1	9 (0.4)	0	9 (4.7)	—	—
s7a1	9 (0.4)	0	9 (4.7)	—	—
s7a2	9 (0.4)	0	9 (4.7)	—	—
s8a1	59 (2.5)	50 (2.4)	9 (4.7)	—	—
s8a2	57 (2.4)	48 (2.2)	9 (4.7)	—	—
s8a3	54 (2.3)	44 (2)	9 (4.7)	5.43 (1)	.02

^a^The percentage in brackets is calculated in relation to the initial number of participants per group.

^b^Not applicable.

In addition, based on the *χ*^2^ test of association and Cramér value, there is a significant difference between basic and premium users in terms of the engagement rate at the end of step 0 (s0a4; n=2331; *χ*^2^_1_=7.8; *P*=.005), the beginning of step 1 (s1a1; n=2331; *χ*^2^_1_=68.1; *P*≤.001), and the end of the preparation program (S8a3; n=2331; *χ*^2^_1_=5.43; *P*=.02). Premium users were more likely than basic users to finish the last activity of step 0 (s0a4; premium=91.5% vs basic=85.2%), start the new step (s1a1; premium=26.9% vs basic=9.7%), and complete all the program’s activity until the definition of the quit date (S8a3; premium=4.7% vs basic=2%).

#### The Impact of the Program Completion on Users Who Finished the Program on Motivation Levels Toward Quitting

From an initial sample of 2331 ecological users, only 57 (2.4%) completed activity s8a2, which assesses the motivation levels regarding WAR scale. Table 4 presents the WAR scale and subscales scores from the beginning (s0a2) to the end of the program (s8a2) of the 2 users’ groups who finished the program (57/2331, 2.4%) as well as the Mann-Whitney score.

**Table 4:** Description of the Total Willingness, Ability, and Readiness To Quit (WAR) scale scores and subscales at the start (s0a2) and end (s8a2) of the 9s-Kwit’s program (n=57).

WAR scale			Total sample				Basic sample (n=48)				Premium sample (n=9)					Mann-Whitney test^a^					*P* value
			Values, mean (SD)		Values, median		Values, mean (SD)		Values, median		Values, mean (SD)		Values, median		U			Cohen *d*^b^			
**Willingness**																					
	s0a2	7.46 (1.89)		8.00		7.40 (1.87)		7.00		7.78 (2.11)		8.00		188			0.12		.54		
	s8a2	7.75 (1.91)		8.00		7.63 (1.94)		8.00		8.44 (1.67)		9.00		160			0.26		.21		
**Ability**																					
	s0a2	5.33 (1.89)		5.00		5.02 (1.71)		5.00		7.00 (2.06)		7.00		94			0.56		.007		
	s8a2	6.25 (2.14)		6.00		5.98 (2.09)		6.00		7.67 (1.94)		7.00		118			0.45		.03		
**Readiness**																					
	s0a2	6.30 (1.99)		6.00		6.23 (1.86)		6.00		6.67 (2.69)		6.00		201			0.06		.75		
	s8a2	6.86 (2.06)		7.00		6.77 (2.05)		6.50		7.33 (2.12)		7.00		184			0.15		.48		
**Total**																					
	s0a2	6.36 (1.37)		6.33		6.22 (1.22)		6.00		7.15 (1.68)		6.33		190			0.12		.57		
	s8a2	6.95 (1.68)		6.67		6.79 (1.64)		6.67		7.81 (1.73)		7.60		144			0.32		.13		

^a^*P* values for the Mann-Whitney test were statistically significant at *P*<.05.

^b^Cohen *d* effect size indicating the magnitude of the effect, where a small effect is approximately *d*=0.2, a medium effect is *d*=0.5, and a large effect is *d*=0.8.

To estimate the program’s impact on the motivation levels of the program completion, Student *t* tests were conducted within each group.

At the beginning of the program, users who finished (57/2331, 2.4%) reported feeling, on average, highly willing to quit smoking (7.46/10), moderately confident in their abilities to do so (5.33/10), and moderately ready to start the quitting journey (6.30/10). A Mann-Whitney *U* test was performed to assess whether scores on the WAR scale and its subscales differed significantly between users of each group both at the beginning and at the end of the program. The results indicated that only subscale Ability differs significantly between both groups’ samples (basic and premium), with a moderate effect size (*U*=6.86; *z* score=118; *P*=.02; *d*=0.45) before (s0a2) and after the program completion (s8a2). Users who had the app’s premium version reported feeling significantly more capable of smoking cessation than users who had the basic version before (basic=5.02; premium=7) and after (basic=5.98; premium=7.33) the completion of the preparation program.

Premium users who finished 9s-Kwit’s program reported, with a high effect size, significantly higher scores than at the beginning of the program on the Willingness subscale (*t*_8_=2.83; *P*=.02; Cohen *d*=0.9) and the total score of the WAR scale (*t*_8_=3.16; *P*=.01; Cohen *d*=1.05). Conversely, users who completed the basic version of the program (4 steps) reported, with moderate effect size, significantly higher end-of-program scores in the Ability subscale (t_47_=3.17; *P*=.003; Cohen *d*=0.46) and a total score of the WAR scale (t_47_=2.92; *P*=.005; Cohen *d*=0.42), and with low effect size, significantly higher end-of-program scores in the Readiness subscale (t_47_=1.857; *P*=.07; Cohen *d*=0.26).

## Discussion

### Overview

This study aimed, in an ecological context, to explore the engagement rate of a 9s-Kwit’s program on a mobile app (Kwit app); to examine some moderating agents that could contribute to the engagement rate we observed; and to estimate the impact of the program on the motivation level among users who finished the program. An ecological context refers to research conducted in natural, real-world settings as opposed to controlled laboratory environments. This approach is crucial for several reasons. First, it embeds our investigation in real-life scenarios where participants interact with the Kwit app as part of their daily routines. This allows us to gain authentic insights into user behaviors, app engagement levels, and the program’s impact, mirroring genuine use patterns more accurately than laboratory conditions could. Such insights are invaluable for designing interventions that are not only effective in theory but also practical and beneficial for users in their real-world context.

### Principal Findings

#### Overview

A total of 2331 users, all of whom were active smokers without a set quit date, started the 9s-Kwit’s program. Most users (2142/2331, 91.89%) opted for the basic version of the program, whereas 8% (n=189) selected the premium one. Only 2.3% (54/189) of the initial sample reached the last step of the program (s8), which consisted of defining a quit date.

Notably, there was a marked difference in completion rates between the 2 versions: 4.7% (9/189) of premium users finished all program steps, which was higher than the 2.95% (44/2142) completion rate observed in the basic version. This significant disparity in engagement rates (n=2331; *χ*^2^_1_=5.4; *P*=.02) highlights a greater commitment among premium users, despite them having more steps to complete.

To better understand the attrition phenomenon, we monitored user engagement at each program activity and observed 3 prominent dropouts. The first and most significant was observed between the last activity of step 0 (s0a4) and the beginning of step 1 (s1a1), with higher attrition rate among basic users compared to premium users (Table 2). The second dropout was observed in the basic user sample between s2a2 and s8a1 (–4.2%), and the third dropout was within the premium sample from activity s4a2 to s5a1 (–12.5%). These dropout patterns can be attributed to 3 main factors: (1) specific user characteristics (ie, areas of motivation and perceived content relevance), (2) the specific attributes of the app’s user experience and the cognitive effort required to engage with different activities, and (3) typical attrition rates for smoking cessation interventions and the regular use of mHealth solutions.

#### Specific User Characteristics at the Beginning of the Program

In this study, data collection was confined to what was essential for the program’s use, focusing on motivation areas and perceived content relevance, without collecting demographic information or insights into users’ health and technology literacy. Following the rationale that user motivation to quit and user satisfaction with the app determines user engagement, higher engagement rates were anticipated. Only 26.9% (56/2142) of premium users and 9.7% (209/189) of basic users progressed beyond the initial stage of the program (from s0a4 to s1a2). Notably, while >70% (1668/2331) of both groups rated the content as “understandable and relevant,” there was a significant perceived relevance gap, with premium users rating the app content more relevant by >10 points compared to basic users, as detailed in Table 2. This discrepancy suggests that content relevance may play a substantial role in influencing engagement levels, particularly among different user groups.

Significant differences were also observed in the initial sample (N=2331) between the 2 groups in all motivational measures: the reasons for quitting smoking, the WAR scale, and the internalization level to quit smoking. Premium users, who were more likely to choose wellness and family planning as their primary reason for quitting, also showed greater willingness, ability, and readiness to quit smoking at baseline compared to basic users, who were primarily motivated by financial savings. Interestingly, there is a difference between the willingness to quit and the user’s self-perception of their ability and readiness to quit. On average, both groups reported high levels of willingness to quit smoking (premium=7.78; basic=7.40) but a moderate perception of their ability (premium=7; basic=5.02) and readiness (premium=6.67; basic=6.23) to do so. In addition, higher levels of internalization were observed in the premium sample with fewer incomplete responses (premium=15.9%; basic=9%) and lower amotivation profile (premium=1.6%; basic=3.6%). They were more represented in profiles of identified (premium=9%; basic=6.4%) and intrinsic motivation (premium=28%; basic=20.4%).

In summary, while both groups reported being highly willing to quit smoking, premium users reported feeling more confident and prepared to start their smoking cessation process, more driven by internalized motivation and reasons to quit (ie, well-being vs savings), and more committed from the early stages of the program.

#### The Specific Attributes of the Mobile App User Experience

Despite being user centered and clinically guided, the 9s-Kwit’s program design might contribute to user disengagement at specific points: (1) for both versions in the transition between the last activity of step 0 (s0a4) and the beginning of step 1 (s1a1); (2) for the basic version, between step 2 (s2a2) and step 8 (s8a1); and (3) for the premium version, between step 4 and step 5. The AIM-ACT framework helps explain this attrition: emotional and motivational mismatches (AIM) and attentional or contextual misalignments (ACT) could hinder user engagement, especially during key step changes. This suggests the need for aligning app activities more closely with users’ affective states, goals, and contextual realities to reduce dropout rates [27].

The first problem arises in the transition of the app between the dashboard and the activity screen (Multimedia Appendix 2). When users complete all activities in a step, they are redirected to the dashboard, where an animation (an opening of a small padlock) indicates the next available step. This transition can create friction rather than facilitate smooth progression, potentially discouraging motivated users from starting new activities. Alternative design elements, such as more prominent visual cues (eg, a red circle above the start step) or direct navigation to the next activity screens, could improve user engagement. Second, for basic version users, the dashboard could be improved by more clearly indicating accessible steps, perhaps by visually distinguishing available and unavailable steps, thus reducing confusion and friction in progression. For example, it would have been possible to slightly blur the steps to which they did not have full access to highlight the user’s path and decrease friction between step 2 and step 8.

The third issue involves the cognitive load of certain activities, particularly those that require more active engagement, such as reading or logging cravings for a long period. This could be the case for the dropout in between step 4 (s4) and step 5 (s5); 3 types of exercises were proposed in s4 to be performed for 24 hours, setting goals for craving management. The design of these activities, including their duration and nature, might impact users’ perception of the effort-to-reward ratio as insufficient to continue engaging with the app program.

In general, using any game element, such as achieving certain milestones, will encourage learning new skills and knowledge by making learning more enjoyable and engaging and by enhancing self-esteem and motivation, which are highly associated with long-term smoking cessation [25,38]. The version of the Kwit app used in this review represents the first stage of an evolving series of modifications. This baseline version was designed to establish a solid evidence-based core, filling a gap where many mobile smoking cessation apps have lacked a scientific foundation [10,39].

#### The Standardized Attrition Rate for Smoking Cessation and mHealth Solutions

The dropout rate observed in this study could be because of the nature of tobacco use disorders and the selection criteria. Tobacco use disorders are well studied, with various effective treatments available; however, they typically exhibit dropout rates ranging from 10.8% to 77% in participants who had already defined a quit date as part of the selection criteria [20]. According to model developed by Prochaska and Prochaska [17], our participants would be at a lower stage because they would be just in the preparation phase.

Moreover, attrition is a known challenge in mHealth, with an average dropout rate of 43%, with higher rates in research under ecological conditions (98%) and lower rates in randomized controlled trials (up to 30%) [21].

In the case of studies on smoking cessation apps, the reporting of quit rates has yet to be standardized and is not always reported. This threatens the validity of some studies by introducing selection bias and making it difficult to compare the effect of such apps [11]. For instance, dropout rates in such studies range from 19.3% at 28 days [22] to 39.5% at 87.3 days of use [23] in controlled settings with English-speaking participants, while studies in ecological settings with French-speaking participants report rates of 65% at 30 days and 80% at 90 days of use [39] with a 1-year data collection period (vs less than a month for our study).

#### The Impact of the Program Completion on Users’ Motivation Levels Toward Quitting

To estimate the program’s impact on the motivation level, the analysis was based on 57 users who completed the activity s8a2 of the program: 2.2% of the users from the basic version (n=48) and 4.7% from the premium version (n=9). At the beginning of the program, both groups reported high levels of willingness to quit smoking, with an average willingness score of 7.46 out of 10. However, they reported only moderate levels of ability and preparedness, with average scores of 5.33 out of 10 for their perceived ability to quit and 6.30 out of 10 for their readiness to begin the quitting process.

After the program completion, substantial improvements in the WAR total score were observed in both groups, with different areas of improvement for each group. The premium users improved their willingness levels, whereas users with the basic version benefited in the ability and readiness category. Given the difficulty of assessing the effect of each individual component, we can only hypothesize about the program content’s effect on WAR scores. It is worth noting that throughout her work, Michie has linked several techniques based on behavioral theories to different behavior change as smoking cessation to the point that there is now an ontology and a whole system that would allow this question to be answered in future studies [40,41]. Therefore, the content of the 9s-Kwit’s program was mainly described through the taxonomy developed by Michie et al [32,33].

Given the intricate challenge of isolating and evaluating the effect of individual components within the program, our analysis regarding its impact on WAR scale scores must be approached with caution. As such, we posit that the program’s influence on users’ sense of competence and readiness likely stems from the implementation of various CBT techniques previously recognized as efficacious for smoking cessation in personal intervention settings [42].

Extensive research into personal interventions across varied demographics and environments has pinpointed specific CBT techniques—and their synergies—to be instrumental in elevating smoking cessation success rates. The 9s-Kwit’s program incorporates these proven techniques, including goal setting for behaviors and outcomes, using problem-solving strategies, revisiting and refining behavioral goals, fostering commitment, delivering feedback on behavioral outcomes, enabling behavior substitution, facilitating behavioral practice and rehearsal, and reconfiguring both physical and social contexts to bolster behavior change efforts [42]. These components are strategically combined to amplify the probability of cessation among users.

Moreover, the program integrates principles from acceptance and commitment therapy right from the outset, urging users to align with their core values, such as family, well-being, or personal health. Using metaphors, these values are presented as guiding beacons during moments of temptation (cravings) [43]. The “plus” button feature distinctively separates sensations from actions, offering various strategies such as the 4Ds (Delay, Deep breath, Drink water, Do something else) and meditation to manage cravings effectively [44]. In the second stage, the Horn scale demystifies the multifaceted reasons behind smoking habits, proposing tailored strategies for each [6]. Culminating in stage 8, the program prompts users to confront their quitting fears, providing customized solutions to navigate these apprehensions successfully and asking them to set a quit date.

These results suggest that Kwit’s preparation program can address ambivalence by increasing willingness to change, self-confidence, and readiness to quit among its users, especially those who felt less able to do so (users with the basic version). Self-efficacy or confidence in one’s abilities has been identified as a determinant in long-term abstinence [45].

### Strengths and Limitations

#### Strengths

The strength of this type of study is simultaneously one of its major limitations; being observational in an ecological context, it offers a unique “snapshot” of real-life behavior of a group of users of a mobile smoking cessation app, providing insight into how its use may impact motivation outcomes. This approach not only sheds light on the “science of attrition” by recognizing when intervention dropouts occur but also aids in identifying real-life adoption challenges, thereby facilitating the generation of hypotheses to enhance health care treatment adherence, particularly with the evolution of commercial mHealth apps [21,29]. To the best of our knowledge, only 2 other studies have explored using mHealth tools [46,47] to treat smokers who are ambivalent about quitting, and these studies focused on French-speaking smokers. This is the third study to examine this topic within an ecological context (ie, popular mobile apps) and with a large sample size [39,48].

#### Limitations

The low generalizability of the findings was a limitation for several reasons, outlined in the following sections.

#### Lack of Individual-Level Measures

The absence of detailed individual-level data, particularly on smoking habits, dependency levels, and mental health issues such as anxiety and depression, is a significant limitation. These factors are known to be associated with lower adherence to treatment [48,49]. In additional, the study did not collect detailed demographic information, such as age and gender, before the use of the 0-step preparation program. Conducted within an ecological context and adhering to General Data Protection Regulation, the study’s data collection was limited to what was essential for app functionality, excluding pre-use demographic details.

#### Sample Size and Demographics

The study’s sample is limited to iOS users, excluding Android users, who may differ in terms of quit attempts, quit date settings [50], and sociodemographics [51]. Given the global market share and diverse user base of Android, this limitation means the findings may not fully represent the broader population of smartphone users. The decision to focus on iOS users was driven by specific constraints at the time of the research design, making the results more reflective of the iOS user group.

#### Short Recruitment and Follow-Up Duration

The short duration of the recruitment phase and follow-up period is another limitation. Smoking cessation is a complex process often involving cycles of relapse and recovery. A brief follow-up may only capture a snapshot of this process, potentially overlooking critical data on sustained abstinence or long-term relapse rates. It may also fail to adequately assess dropout rates and reasons for disengagement, which are crucial for understanding and improving app efficacy.

#### Small Sample Size of Premium Users

The explanatory power of the study is also limited by the small sample size of premium users who completed the 9s-Kwit program. Although similar attrition rates have been reported in other ecological studies [39,48], the small number of premium users reduces the generalizability of the findings.

### Future Perspectives

To address all these limitations and investigate user engagement over extended periods, we have established a new protocol in collaboration with the University Paris Nanterre, registered in ClinicalTrials.gov (NCT05318651). This initiative aims to provide a more comprehensive understanding of the program’s impact over time.

Despite the limitations outlined, studies such as ours are critical for shedding light on 1 stage of the mHealth app design process. This research provided the Kwit design team with critical early insights that allowed for rapid iterative adjustments through A/B testing before launching the program on the Android platform and localizing it for different language audiences. As for 2024, the newly developed program integrates game-like features (awarding users points for successfully completing activities, offering different levels, and allowing for avatar customization), educational reading materials related to the topic of each step of the program, and a community design that was not previously accessible. It also introduces educational reading materials and a community design, which were not previously available on the platform. It remains to be seen how this proactive and responsive approach to app design not only optimizes user engagement but also avoids significant costs. It underscores the critical role of early and ongoing user feedback in refining mHealth interventions to ensure that they are both effective and engaging.

Rather than focusing on increasing abstinence as a stand-alone intervention, new studies should aim to improve the use of these and other interventions (as a complementary treatment rather than a comparative one) [48] and consider dropout rates as a measure of intervention quality. A meager retention rate highlights the current inability to satisfy users’ expectations and needs of those seeking to quit [21]. To increase the duration and intensity of app use, qualitative studies are needed to obtain a user-centered perspective. In addition, small trials with users can help guide decision-making on design tweaks or new features. In contrast, through a quasi-experimental observational study based on the technology acceptance model, it would be possible to identify the determinants of engagement with a smoking cessation app [31].

### Conclusions

This study on the first version of the 9s-Kwit’s program provides crucial insights into the challenges and potential of mHealth apps in facilitating smoking cessation efforts. Despite a modest completion rate—with only 2.3% (57/2331) of the participants reaching the program’s final step—the data unveil a significant difference in engagement levels between users of the basic and premium versions.

At the program’s onset, participants displayed a strong willingness to quit, yet their self-assessed ability and readiness were only moderate. Notably, premium users reported feeling more capable of quitting than basic users—a sentiment that not only persisted but also intensified by the program’s end. Subsequent evaluations indicated significant enhancements in willingness, ability, and readiness to quit across both user groups, with premium users showing an increased willingness and basic users experiencing considerable gains in self-assessed ability and readiness to quit.

These results imply that, despite the study’s limitations, the 9s-Kwit’s program may address user ambivalence, thereby enhancing willingness, confidence, and readiness to quit, particularly among those who initially felt less capable. Such improvements in self-efficacy are vital, as they play a pivotal role in achieving long-term smoking cessation success [45].

Ultimately, this research adds to the broader discourse on digital health interventions, underscoring the critical importance of adaptive design and user-centric approaches in developing effective mHealth solutions [24,26,27]. The observed attrition patterns highlight the intricate interaction between user characteristics, the cognitive demands of the app, and the inherent challenges of smoking cessation. This emphasizes the essential need for early, iterative design processes that are informed by user feedback.

However, the study’s conclusions must be approached with caution because of limitations such as the small sample size, the exclusive focus on iOS users, and the lack of comprehensive demographic data. By acknowledging these limitations, we have initiated a new research protocol with the University of Paris Nanterre (NCT05318651) to extend our investigation. This study aims to assess the long-term impact of the 9s-Kwit’s program on a more diverse and extensive participant base.

## Appendix

Multimedia Appendix 1 User flow for accessing Kwit’s smoking cessation preparation program.

Multimedia Appendix 2 The 9-step preparation program dashboard screen.

Multimedia Appendix 3 Presentation of the scales.

## Abbreviations

9s-Kwit’s program: Kwit’s 9-step preparation program for smoking cessation
CBT: cognitive behavioral therapy
F-SCMS: French Smoking Cessation Motivation Scale
mHealth: mobile health
VAS: visual analog scale
WAR: Willingness, Ability, and Readiness to Quit
WHO: World Health Organization

## References

[1] (2021). WHO report on the global tobacco epidemic 2021: addressing new and emerging products. World Health Organization.

[2] Cogordan C, Quatremère G, Andler R, Guignard R, Richard JB, Nguyen-Thanh V (2020). [Dialogue between general practitioner and patient regarding tobacco and alcohol consumption, from the patient's standpoint]. Rev Epidemiol Sante Publique.

[3] Donny EC, Denlinger RL, Tidey JW, Koopmeiners JS, Benowitz NL, Vandrey RG, al’Absi M, Carmella SG, Cinciripini PM, Dermody SS, Drobes DJ, Hecht SS, Jensen J, Lane T, Le CT, McClernon FJ, Montoya ID, Murphy SE, Robinson JD, Stitzer ML, Strasser AA, Tindle H, Hatsukami DK (2015). Randomized trial of reduced-nicotine standards for cigarettes. N Engl J Med.

[4] Hatsukami DK, Carroll DM (2020). Tobacco harm reduction: past history, current controversies and a proposed approach for the future. Prev Med.

[5] Palmer AM, Toll BA, Carpenter MJ, Donny EC, Hatsukami DK, Rojewski AM, Smith TT, Sofuoglu M, Thrul J, Benowitz NL (2022). Reappraising choice in addiction: novel conceptualizations and treatments for tobacco use disorder. Nicotine Tob Res.

[6] (2014). Arrêt de la consommation de tabac: du dépistage individuel au maintien de l’abstinence en premier recours. Haute Autorité de Santé.

[7] (2022). WHO launches Quit Tobacco App. World Health Organization.

[8] Cambon L, Bergman P, Le Faou A, Vincent I, Le Maitre B, Pasquereau A, Arwidson P, Thomas D, Alla F (2017). Study protocol for a pragmatic randomised controlled trial evaluating efficacy of a smoking cessation e-'Tabac Info Service': ee-TIS trial. BMJ Open.

[9] Brown J, Michie S, Geraghty AW, Yardley L, Gardner B, Shahab L, Stapleton JA, West R (2014). Internet-based intervention for smoking cessation (StopAdvisor) in people with low and high socioeconomic status: a randomised controlled trial. Lancet Respir Med.

[10] Bustamante LA, Gill Ménard C, Julien S, Romo L (2021). Behavior change techniques in popular mobile apps for smoking cessation in France: content analysis. JMIR Mhealth Uhealth.

[11] Bahadoor R, Alexandre JM, Fournet L, Gellé T, Serre F, Auriacombe M (2021). Inventory and analysis of controlled trials of mobile phone applications targeting substance use disorders: a systematic review. Front Psychiatry.

[12] Vilardaga R, Casellas-Pujol E, McClernon JF, Garrison KA (2019). Mobile applications for the treatment of tobacco use and dependence. Curr Addict Rep.

[13] Regmi K, Kassim N, Ahmad N, Tuah NA (2017). Effectiveness of mobile apps for smoking cessation: a review. Tob Prev Cessat.

[14] Whittaker R, McRobbie H, Bullen C, Rodgers A, Gu Y, Dobson R (2019). Mobile phone text messaging and app-based interventions for smoking cessation. Cochrane Database Syst Rev.

[15] Auriacombe M, Fatséas M, Daulouède J, Tignol J (2018). Le craving et nouvelle clinique de l’addiction: une perspective simplifiée et opérationnelle. Annales Médico-Psychologiques, Revue Psychiatrique.

[16] Hatsukami DK, Luo X, Jensen JA, al'Absi M, Allen SS, Carmella SG, Chen M, Cinciripini PM, Denlinger-Apte R, Drobes DJ, Koopmeiners JS, Lane T, Le CT, Leischow S, Luo K, McClernon FJ, Murphy SE, Paiano V, Robinson JD, Severson H, Sipe C, Strasser AA, Strayer LG, Tang MK, Vandrey R, Hecht SS, Benowitz NL, Donny EC (2018). Effect of immediate vs gradual reduction in nicotine content of cigarettes on biomarkers of smoke exposure: a randomized clinical trial. JAMA.

[17] Prochaska JO, Prochaska JM (2016). Changing to Thrive: Using the Stages of Change to Overcome the Top Threats to Your Health and Happiness.

[18] Serre F, Fatseas M, Swendsen J, Auriacombe M (2015). Ecological momentary assessment in the investigation of craving and substance use in daily life: a systematic review. Drug Alcohol Depend.

[19] (2003). Adherence to long-term therapies: evidence for action. World Health Organization.

[20] Belita E, Sidani S (2015). Attrition in smoking cessation intervention studies: a systematic review. Can J Nurs Res.

[21] Meyerowitz-Katz G, Ravi S, Arnolda L, Feng X, Maberly G, Astell-Burt T (2020). Rates of attrition and dropout in app-based interventions for chronic disease: systematic review and meta-analysis. J Med Internet Res.

[22] Ubhi HK, Michie S, Kotz D, Wong WC, West R (2015). A mobile app to aid smoking cessation: preliminary evaluation of SmokeFree28. J Med Internet Res.

[23] Marler JD, Fujii CA, Utley DS, Tesfamariam LJ, Galanko JA, Patrick H (2019). Initial assessment of a comprehensive digital smoking cessation program that incorporates a mobile app, breath sensor, and coaching: cohort study. JMIR Mhealth Uhealth.

[24] Sobolev M, Anand A, Dziak JJ, Potter LN, Lam CY, Wetter DW, Nahum-Shani I (2023). Time-varying model of engagement with digital self reporting: evidence from smoking cessation longitudinal studies. Front Digit Health.

[25] Rajani NB, Bustamante L, Weth D, Romo L, Mastellos N, Filippidis FT (2023). Engagement with gamification elements in a smoking cessation app and short-term smoking abstinence: quantitative assessment. JMIR Serious Games.

[26] Alkhaldi G, Hamilton FL, Lau R, Webster R, Michie S, Murray E (2016). The effectiveness of prompts to promote engagement with digital interventions: a systematic review. J Med Internet Res.

[27] Nahum-Shani I, Shaw SD, Carpenter SM, Murphy SA, Yoon C (2022). Engagement in digital interventions. Am Psychol.

[28] Bricker JB, Mull KE, Santiago-Torres M, Miao Z, Perski O, Di C (2022). Smoking cessation smartphone app use over time: predicting 12-month cessation outcomes in a 2-arm randomized trial. J Med Internet Res.

[29] Eysenbach G (2005). The law of attrition. J Med Internet Res.

[30] Moore TM, Seavey A, Ritter K, McNulty JK, Gordon KC, Stuart GL (2014). Ecological momentary assessment of the effects of craving and affect on risk for relapse during substance abuse treatment. Psychol Addict Behav.

[31] Bustamante L, Romo L (2022). Use determinants of smoking cessation app. National Institutes of Health National Library of Medicine.

[32] Michie S, Hyder N, Walia A, West R (2011). Development of a taxonomy of behaviour change techniques used in individual behavioural support for smoking cessation. Addict Behav.

[33] Michie S, Wood CE, Johnston M, Abraham C, Francis JJ, Hardeman W (2015). Behaviour change techniques: the development and evaluation of a taxonomic method for reporting and describing behaviour change interventions (a suite of five studies involving consensus methods, randomised controlled trials and analysis of qualitative data). Health Technol Assess.

[34] Pisinger C, Aadahl M, Toft U, Jørgensen T (2011). Motives to quit smoking and reasons to relapse differ by socioeconomic status. Prev Med.

[35] McCaul KD, Hockemeyer JR, Johnson RJ, Zetocha K, Quinlan K, Glasgow RE (2006). Motivation to quit using cigarettes: a review. Addict Behav.

[36] Gentry S, Craig J, Holland R, Notley C (2017). Smoking cessation for substance misusers: a systematic review of qualitative studies on participant and provider beliefs and perceptions. Drug Alcohol Depend.

[37] Bustamante LA, Romo L (2022). Validation of the French smoking cessation motivation scale with French smokers using a mobile app for smoking cessation. Eur J Investig Health Psychol Educ.

[38] Rajani NB, Mastellos N, Filippidis FT (2021). Impact of gamification on the self-efficacy and motivation to quit of smokers: observational study of two gamified smoking cessation mobile apps. JMIR Serious Games.

[39] Affret A, Luc A, Baumann C, Bergman P, Le Faou AL, Pasquereau A, Arwidson P, Alla F, Cambon L (2020). Effectiveness of the e-Tabac Info Service application for smoking cessation: a pragmatic randomised controlled trial. BMJ Open.

[40] Marques MM, Wright AJ, Corker E, Johnston M, West R, Hastings J, Zhang L, Michie S (2023). The behaviour change technique ontology: transforming the behaviour change technique taxonomy v1. Wellcome Open Res.

[41] Michie S, West R, Finnerty AN, Norris E, Wright AJ, Marques MM, Johnston M, Kelly MP, Thomas J, Hastings J (2020). Representation of behaviour change interventions and their evaluation: development of the upper level of the behaviour change intervention ontology. Wellcome Open Res.

[42] Black N, Johnston M, Michie S, Hartmann-Boyce J, West R, Viechtbauer W, Eisma MC, Scott C, de Bruin M (2020). Behaviour change techniques associated with smoking cessation in intervention and comparator groups of randomized controlled trials: a systematic review and meta-regression. Addiction.

[43] Bricker JB, Mull KE, Kientz JA, Vilardaga R, Mercer LD, Akioka KJ, Heffner JL (2014). Randomized, controlled pilot trial of a smartphone app for smoking cessation using acceptance and commitment therapy. Drug Alcohol Depend.

[44] Maglione MA, Maher AR, Ewing B, Colaiaco B, Newberry S, Kandrack R, Shanman RM, Sorbero ME, Hempel S (2017). Efficacy of mindfulness meditation for smoking cessation: a systematic review and meta-analysis. Addict Behav.

[45] Rajani NB, Mastellos N, Filippidis FT (2021). Self-efficacy and motivation to quit of smokers seeking to quit: quantitative assessment of smoking cessation mobile apps. JMIR Mhealth Uhealth.

[46] McClure JB, Heffner JL, Krakauer C, Mun S, Klasnja P, Catz SL (2023). Feasibility, acceptability, and potential impact of a novel mHealth app for smokers ambivalent about quitting: randomized pilot study. JMIR Mhealth Uhealth.

[47] Houston TK, Chen J, Amante DJ, Blok AC, Nagawa CS, Wijesundara JG, Kamberi A, Allison JJ, Person SD, Flahive J, Morley J, Conigliaro J, Mattocks KM, Garber L, Sadasivam RS (2022). Effect of technology-assisted brief abstinence game on long-term smoking cessation in individuals not yet ready to quit: a randomized clinical trial. JAMA Intern Med.

[48] Etter JF, Khazaal Y (2022). The stop-tabac smartphone application for smoking cessation: a randomized controlled trial. Addiction.

[49] Hock ES, Franklin M, Baxter S, Clowes M, Chilcott J, Gillespie D (2023). Covariates of success in quitting smoking: a systematic review of studies from 2008 to 2021 conducted to inform the statistical analyses of quitting outcomes of a hospital-based tobacco dependence treatment service in the United Kingdom. NIHR Open Res.

[50] Ubhi HK, Kotz D, Michie S, van Schayck OC, West R (2017). A comparison of the characteristics of iOS and Android users of a smoking cessation app. Transl Behav Med.

[51] (2014). Smartphones: so many apps, so much time. The Nielsen Company.

