# Supplementary figures and images for: Feasibility and Engagement of a Mobile App Preparation Program (Kwit) for Smoking Cessation in an Ecological Context: Quantitative Study
Source: JMIR Mhealth Uhealth. 2024 Oct 2;12:e51025. doi: 10.2196/51025 (PMC11483257; doi:10.2196/51025)

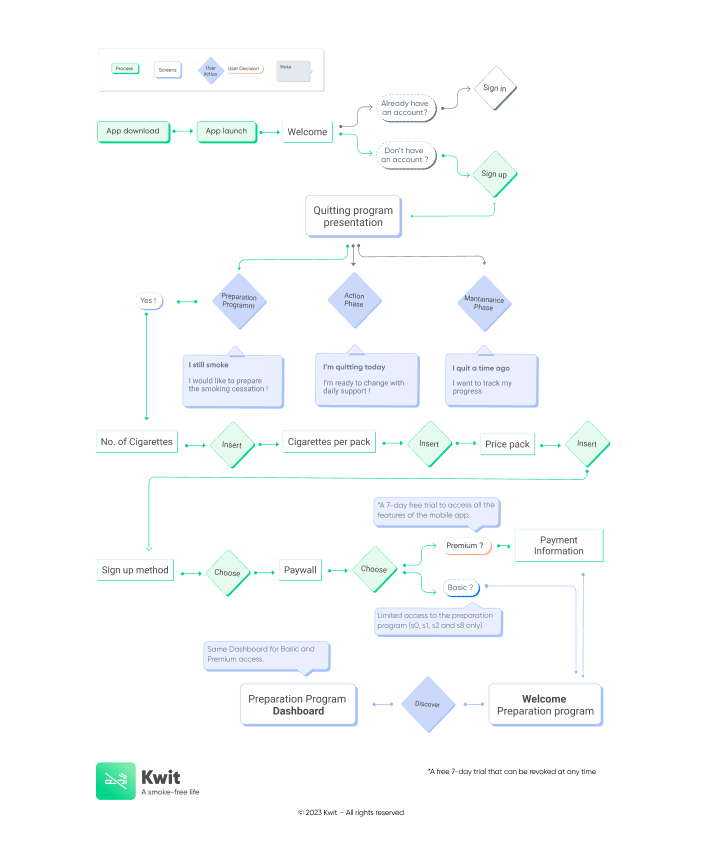

Supplement: Multimedia Appendix 1 [file mhealth_v12i1e51025_app1.png]

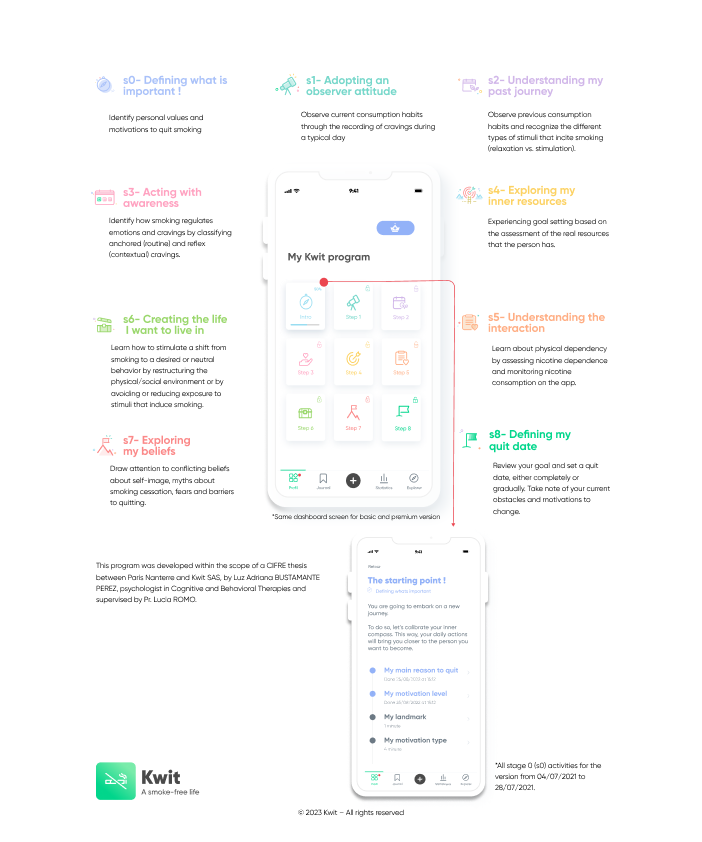

Supplement: Multimedia Appendix 2 [file mhealth_v12i1e51025_app2.png]

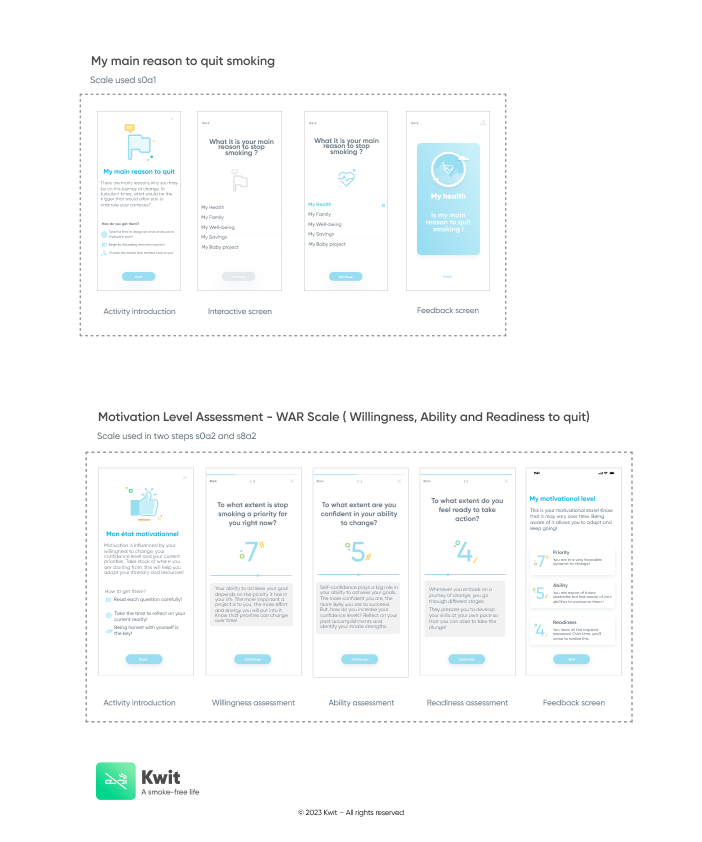

Supplement: Multimedia Appendix 3 [file mhealth_v12i1e51025_app3.png]
